# Alleviation of High-Fat Diet-Induced Fatty Liver Damage in Group IVA Phospholipase A_2_-Knockout Mice

**DOI:** 10.1371/journal.pone.0008089

**Published:** 2009-12-01

**Authors:** Hiromi Ii, Naoki Yokoyama, Shintaro Yoshida, Kae Tsutsumi, Shinji Hatakeyama, Takashi Sato, Keiichi Ishihara, Satoshi Akiba

**Affiliations:** 1 Department of Pathological Biochemistry, Kyoto Pharmaceutical University, Yamashina-ku, Kyoto, Japan; 2 Novartis Institutes for BioMedical Research, Tsukuba, Japan; Mayo Clinic, United States of America

## Abstract

Hepatic fat deposition with hepatocellular damage, a feature of non-alcoholic fatty liver disease, is mediated by several putative factors including prostaglandins. In the present study, we examined whether group IVA phospholipase A_2_ (IVA-PLA_2_), which catalyzes the first step in prostanoid biosynthesis, is involved in the development of fatty liver, using IVA-PLA_2_-knockout mice. Male wild-type mice on high-fat diets (20% fat and 1.25% cholesterol) developed hepatocellular vacuolation and liver hypertrophy with an increase in the serum levels of liver damage marker aminotransferases when compared with wild-type mice fed normal diets. These high-fat diet-induced alterations were markedly decreased in IVA-PLA_2_-knockout mice. Hepatic triacylglycerol content was lower in IVA-PLA_2_-knockout mice than in wild-type mice under normal dietary conditions. Although high-fat diets increased hepatic triacylglycerol content in both genotypes, the degree was lower in IVA-PLA_2_-knockout mice than in wild-type mice. Under the high-fat dietary conditions, IVA-PLA_2_-knockout mice had lower epididymal fat pad weight and smaller adipocytes than wild-type mice. The serum level of prostaglandin E_2_, which has a fat storage effect, was lower in IVA-PLA_2_-knockout mice than in wild-type mice, irrespective of the kind of diet. In both genotypes, high-fat diets increased serum leptin levels equally between the two groups, but did not affect the serum levels of adiponectin, resistin, free fatty acid, triacylglycerol, glucose, or insulin. Our findings suggest that a deficiency of IVA-PLA_2_ alleviates fatty liver damage caused by high-fat diets, probably because of the lower generation of IVA-PLA_2_ metabolites, such as prostaglandin E_2_. IVA-PLA_2_ could be a promising therapeutic target for obesity-related diseases including non-alcoholic fatty liver disease.

## Introduction

Phospholipase A_2_ (PLA_2_) is an enzyme that catalyzes the hydrolysis of glycerophospholipids at the *sn*-2 position, generating free fatty acid (FFA) and lysophospholipid. Among the more than twenty isozymes of mammalian PLA_2_, group IVA PLA_2_ (IVA-PLA_2_) is a key enzyme responsible for the release of arachidonic acid, a precursor of prostaglandins (PGs) [1]. Our recent study of IVA-PLA_2_-knockout mice fed normal chow diets demonstrated that decreases in hepatic triacylglycerol (TG) content and the size of epididymal adipocytes are observed with a lower serum level of PGE_2_ compared with wild-type mice. This suggests that the circulating level of PGE_2_ is related to the levels of intracellular TG in the liver and adipose tissues [2]. Previous reports showed that the stimulation of rat hepatocytes with PGE_2_ and the administration of PGE_2_ to rats induce increases in TG level in the cells and the liver, respectively [3], [4]. Similarly, 15-deoxy-PGJ_2_ endogenously generated in mature 3T3-L1 adipocytes has been shown to be responsible for the storage of TG [5]. Considering these findings, it is possible that IVA-PLA_2_ mediates fat deposition in the liver and adipose tissues through the generation of PGs.

The accumulation of hepatic TG is a major symptom of alcoholic liver disease. However, hepatic fat deposition resulting from TG accumulation has also been found in the absence of alcohol abuse. Such fatty liver progresses to non-alcoholic fatty liver disease (NAFLD) followed by the development of non-alcoholic steatohepatitis and cirrhosis [6]. NAFLD is closely associated with abdominal obesity, a feature of metabolic syndrome [7]. Obesity from overeating high-fat (HF) and/or high-caloric foods is characterized by an abnormal increase in adipose tissue mass mainly resulting from the excessive storage of TG within adipocytes. The state of obesity is accompanied by changes in the expression and secretion of adipokines including adiponectin and resistin, and by an increased release of FFA from hypertrophied adipocytes. The alterations in the circulating levels of adipokines and FFA are involved in the accumulation of TG causing liver damage [6], [8]. Consequently, factors that modulate intracellular TG levels and/or circulating adipokine levels could be targets for the prevention of fatty liver and the development of NAFLD. Considering our recent finding that hepatic TG content is lower in IVA-PLA_2_-knockout mice fed normal chow diets than in wild-type mice [2], it is possible that a suppression of IVA-PLA_2_ protects against the development of fatty liver under HF dietary conditions.

Taking this into account, in the present study, we examined the possible involvement of IVA-PLA_2_ in the development of fatty liver using IVA-PLA_2_-knockout mice fed HF diets. The current study demonstrated that a deficiency of IVA-PLA_2_ protected mice against HF diet-induced increases in the number of hepatocytes exhibiting cytoplasmic vacuolation and hepatic TG content accompanying liver damage. These findings and further results shown here suggest that IVA-PLA_2_ is involved in the development of fatty liver damage under HF dietary conditions.

## Results

**Figure 1:**
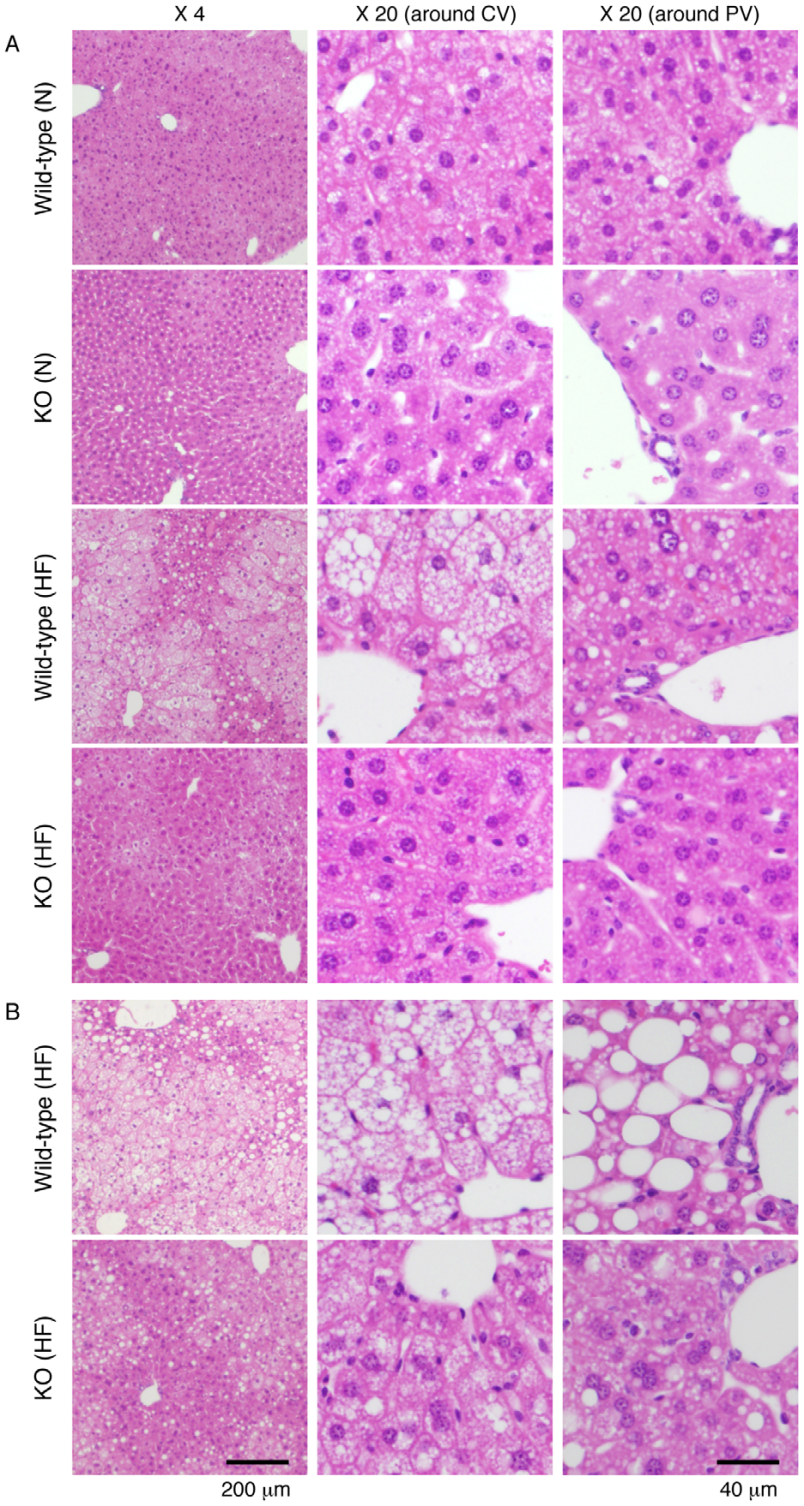
Microscopic views of the liver in wild-type and IVA-PLA_2_-knockout mice. Wild-type and IVA-PLA_2_-knockout (KO) mice were fed normal (N) or high-fat (HF) diets for 8 (A) or 16 (B) weeks and fasted. Representative liver sections stained with hematoxylin-eosin are shown. IVA-PLA_2_ deficiency suppressed the hepatic fat deposition under the HF feeding. CV, central vein; PV, portal vein. Original magnification, ×4 and ×20.

Microscopic views of the liver of wild-type and IVA-PLA_2_-knockout mice fed normal or HF diets for 8 or 16 weeks are shown in Figure 1. The results reveal an apparent cytoplasmic vacuolation of hepatocytes around the central vein in wild-type mice fed HF diets for 8 (Figure 1A) or 16 (Figure 1B) weeks compared with wild-type mice fed normal diets for 8 (Figure 1A) or 16 weeks (data not shown). The degree of hepatic vacuolation increased with the period of feeding. In wild-type mice fed HF diets for 16 weeks, a markedly high level of cytoplasmic vacuolation of hepatocytes around the portal vein was observed compared with that in cells around the central vein (Figure 1B). The vacuolated area was not stained with periodic acid-Schiff, which stains glycogen (data not shown). In contrast to wild-type mice fed HF diets, we found that cytoplasmic vacuolation of hepatocytes around the central and portal veins was strikingly suppressed in IVA-PLA_2_-knockout mice fed HF diets for 8 weeks (Figure 1A). Even after 16 weeks of HF feeding, IVA-PLA_2_-knockout mice exhibited considerably reduced hepatic vacuolation (Figure 1B). Under normal dietary conditions, slight cytoplasmic vacuolation was observed in hepatocytes of wild-type mice; however, this was not the case for IVA-PLA_2_-knockout mice (Figure 1A). This observation in mice fed normal diets was consistent with the results shown in our recent paper [2].

**Figure 2:**
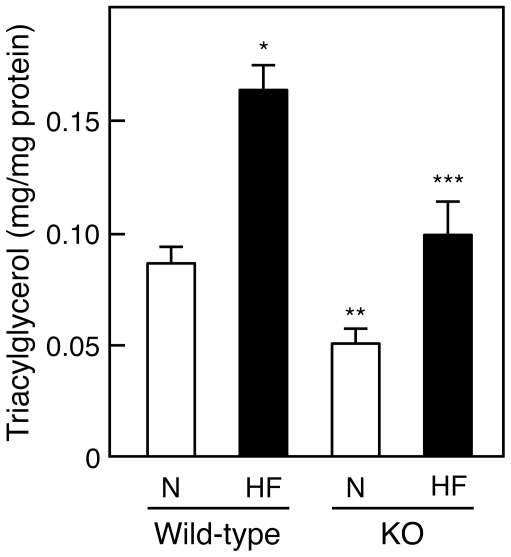
Hepatic triacylglycerol content in wild-type and IVA-PLA_2_-knockout mice. Wild-type and IVA-PLA_2_-knockout mice were fed normal (N) or high-fat (HF) diets for 8 weeks and fasted. Triacylglycerol content in the liver was measured (n = 5–7). IVA-PLA_2_ deficiency suppressed the accumulation of hepatic triacylglycerol under the HF feeding. *P<0.005 versus wild-type mice fed normal diets, **P<0.05 versus wild-type mice fed normal diets, ***P<0.01 versus wild-type mice fed HF diets.

The histological results shown in Figure 1 suggest that a deficiency of IVA-PLA_2_ decreases HF diet-induced fat deposition in the liver, leading us to measure hepatic TG content under HF dietary conditions. Consistent with the histological results, hepatic TG content was apparently greater in wild-type mice fed HF diets for 8 weeks than in wild-type mice fed normal diets, as shown in Figure 2. In IVA-PLA_2_-knockout mice fed normal diets, hepatic TG content was lower than that in wild-type mice, as reported in our recent paper [2]. Although HF diets also increased hepatic TG content in IVA-PLA_2_-knockout mice, the level was significantly lower than that in HF diet-fed wild-type mice, and almost at the level found in normal diet-fed wild-type mice. The degree of the HF diet-induced increase in hepatic TG content was lower in IVA-PLA_2_-knockout mice than in wild-type mice.

**Figure 3:**
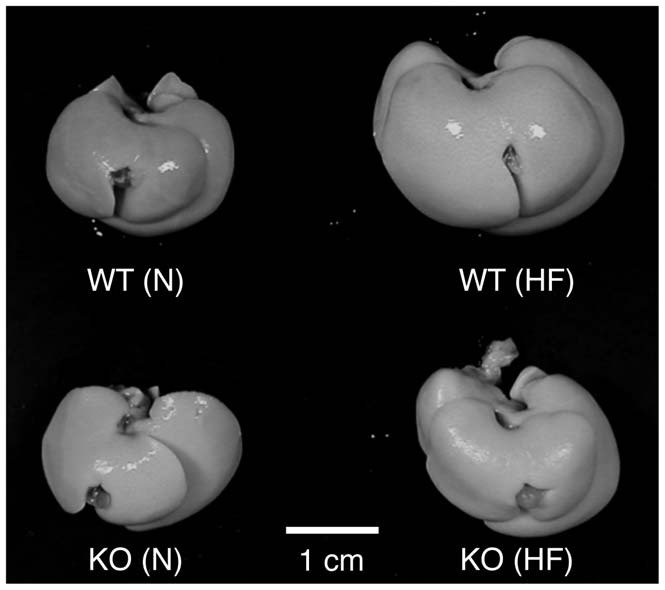
Macroscopic views of the liver in wild-type and IVA-PLA_2_-knockout mice. Wild-type (WT) and IVA-PLA_2_-knockout (KO) mice were fed normal (N) or high-fat (HF) diets for 16 weeks and fasted. Representative photographs of the liver in each mouse are shown. IVA-PLA_2_ deficiency suppressed the hepatic hypertrophy induced by HF feeding.

**Figure 4:**
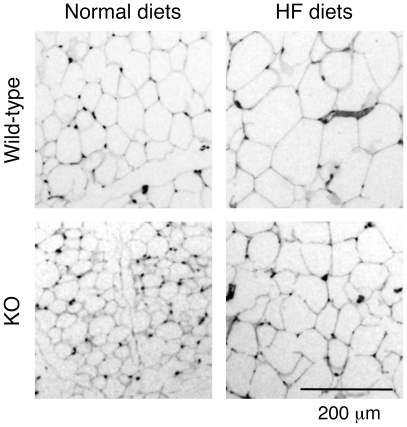
Microscopic views of epididymal fat pads in wild-type and IVA-PLA_2_-knockout mice. Wild-type and IVA-PLA_2_-knockout (KO) mice were fed normal or high-fat (HF) diets for 8 weeks and fasted. Representative epididymal fat pad sections stained with hematoxylin-eosin are shown. Original magnification, ×4. IVA-PLA_2_ deficiency suppressed the expansion of epididymal adipocytes induced by HF feeding.

As shown in Table 1, the weights of the body, liver, and epidermal fat pads increased in wild-type mice fed HF diets for 8 or 16 weeks compared with respective wild-type mice fed normal diets. While no significant difference in these weights was observed between IVA-PLA_2_-knockout mice fed normal and HF diets for 8 weeks, HF feeding for 16 weeks increased these weights. However, these weights in IVA-PLA_2_-knockout mice fed HF diets were lower than those in wild-type mice fed HF diets. Under the dietary conditions, there was no significant difference in the amounts of food intake between the two genotype mice fed HF diets for 16 weeks (wild-type, 2.77±0.18 g/day; IVA-PLA_2_-knockout, 2.90±0.27 g/day, n = 5), or between those fed normal diets for 16 weeks (wild-type, 3.68±0.18 g/day; IVA-PLA_2_-knockout, 3.89±0.10 g/day, n = 7). Consistent with the change in liver weights, macroscopic views of the liver revealed visible hypertrophy in wild-type mice fed HF diets for 16 weeks compared with wild-type mice fed normal diets, as shown in Figure 3. However, such hypertrophy was suppressed in IVA-PLA_2_-knockout mice fed HF diets (Figure 3). Furthermore, the epididymal fat pads were visibly smaller in IVA-PLA_2_-knockout mice fed HF diets than in wild-type mice (data not shown). Similarly, as shown in Figure 4, microscopic views of epididymal fat pads revealed that the adipocytes of epididymal fat pads were larger in wild-type mice fed HF diets for 8 weeks than in wild-type mice fed normal diets. Although HF diets also increased adipocyte size in IVA-PLA_2_-knockout mice, the adipocytes under the HF dietary conditions were smaller in IVA-PLA_2_-knockout mice than in wild-type mice (Figure 4). Similar results were observed after 16 weeks of HF feeding (data not shown).

**Table 1:** Weights of the body, epidermal fat pads, and liver, and serum levels of biochemical parameters under high-fat dietary conditions.

	Body weight (g)	Liver weight (g)	Fat pad weight (g)	FFA (mEq/l)	PL (mg/dl)	LPC (µM)	ALT (U/l)	AST (U/l)
8 weeks of feeding								
WT (N)	23.1±1.3	1.1±0.1	0.4±0.1	0.8±0.1	235±24	377±26	46±5.5	156±24
WT (HF)	31.1±3.9^a^	1.7±0.4	1.3±0.4^a^	0.6±0.1	226±35	455±80	107±25^a^	157±26
KO (N)	25.3±1.4	1.1±0.1	0.2±0.04	0.8±0.1	288±7	418±±39	36±4.0	154±31
KO (HF)	28.5±1.5	1.2±0.04	0.7±0.1	0.6±0.02	275±21	478±53	40±4.9^b^	138±40
16 weeks of feeding								
WT (N)	33.9±2	1.3±0.1	1.1±0.1	0.6±0.1	178±22	407±23	58±19	165±26
WT (HF)	40.8±0.7^a^	2.6±0.4^a^	2.0±0.1^a^	0.6±0.1	361±35^a^	575±33^a^	350±77^a^	366±71^a^
KO (N)	25.2±1.1^c^	1.4±0.1	0.4±0.03^c^	0.8±0.1	173±32	419±18	104±58	215±10
KO (HF)	36.2±2.8	1.9±0.3	1.5±0.5	0.5±0.02	188±8^b^	411±14^b^	87±21^b^	134±21^b^

Wild-type (WT) and IVA-PLA_2_-knockout (KO) mice fed normal (N) or high-fat (HF) diets for 8 or 16 weeks were fasted and subjected to each measurement (n = 4–6). FFA, free fatty acid; PL, phospholipids; LPC, lysophosphatidylcholine; ALT, alanine aminotransferase; AST, aspartate aminotransferase.

a *P*<0.05 versus wild-type mice fed normal diets.

b *P*<0.05 versus wild-type mice fed HF diets.

c *P*<0.005 versus wild-type mice fed normal diets.

**Figure 5:**
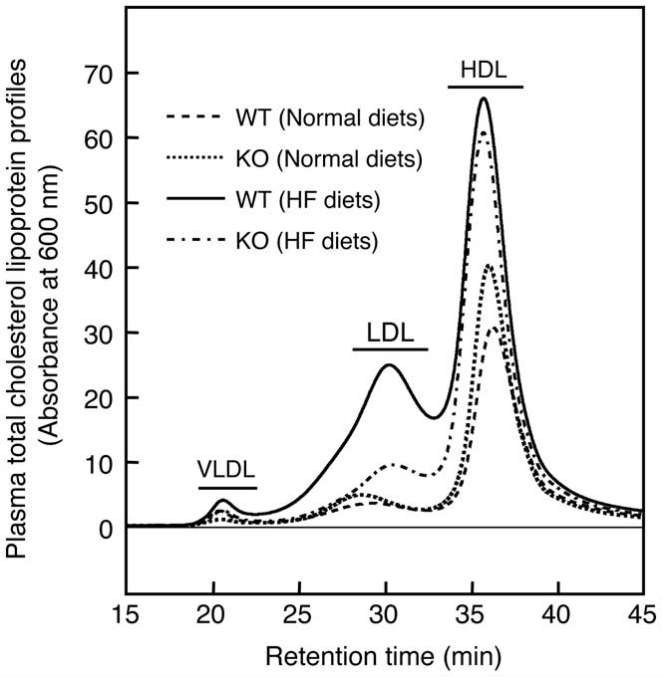
Lipoprotein profiles under HF dietary conditions. Wild-type (WT) and IVA-PLA_2_-knockout (KO) mice were fed normal or HF diets for 16 weeks and fasted. The serum levels of VLDL, LDL, and HDL were analyzed. Each trace represents the mean (n = 4–6). IVA-PLA_2_ deficiency reduced the increased serum level of LDL by HF feeding.

Under the dietary conditions where HF diets taken for 8 weeks induced fat deposition in the liver and adipose tissues of wild-type mice but not IVA-PLA_2_-knockout mice (Figures 1, 2, and 4), no significant change in the serum levels of TG was observed as follows: wild-type mice fed normal diets, 60.8 mg/dl±3.6; wild-type mice fed HF diets, 72.0 mg/dl±3.5; IVA-PLA_2_-knockout mice fed normal diets, 70.8 mg/dl±3.3; IVA-PLA_2_-knockout mice fed HF diets, 75.0 mg/dl±15.5; n = 3–5. Similar results were observed after 16 weeks of feeding (data not shown). Furthermore, there was no difference between the serum levels of FFA for the two genotypes (Table 1). HF diets taken for 8 weeks also did not affect the serum level of glucose (wild-type mice fed normal diets, 135 mg/dl±9; wild-type mice fed HF diets, 118 mg/dl±13; IVA-PLA_2_-knockout mice fed normal diets, 122 mg/dl±15; IVA-PLA_2_-knockout mice fed HF diets, 111 mg/dl±7; n = 4–5). Under our experimental conditions, there were no differences in the serum levels of insulin after 8 weeks of HF feeding as follows: wild-type mice fed normal diets, 0.84 ng/ml±0.21; wild-type mice fed HF diets, 1.14 ng/ml±0.14; IVA-PLA_2_-knockout mice fed normal diets, 1.22 ng/ml±0.10; IVA-PLA_2_-knockout mice fed HF diets, 0.94 ng/ml±0.33; n = 4–5. HF diets taken for 16 weeks also did not affect the serum level of insulin (data not shown). In contrast, while no significant change in the serum level of phospholipids or lysophosphatidylcholine was observed in the two genotype mice fed HF diets for 8 weeks, HF diets taken for 16 weeks increased these serum levels in wild-type mice but not IVA-PLA_2_-knockout mice (Table 1). Consistent with this, the profiles of lipoproteins shown in Figure 5 reveal that wild-type mice fed HF diets for 16 weeks had higher serum levels of low-density lipoprotein (LDL) and high-density lipoprotein (HDL) than wild-type mice fed normal diets, but a change in the serum level of very low-density lipoprotein (VLDL) was scarcely observed. In IVA-PLA_2_-knockout mice fed HF diets, the serum level of LDL but not HDL was reduced compared with those in wild-type mice fed HF diets. Although TG content of these lipoproteins was concomitantly measured, there was no difference between the four groups (data not shown). Furthermore, as shown in Table 2, we confirmed that the serum level of PGE_2_ was significantly lower in IVA-PLA_2_-knockout mice than in wild-type mice under the dietary conditions.

**Table 2:** Serum levels of PGE_2_ in wild-type and IVA-PLA_2_-knockout mice.

	PGE_2_ (ng/ml)
Wild-type mice (N)	4.29±1.05
Wild-type mice (HF)	5.32±1.17
KO mice (N)	0.52±0.08^a^
KO mice (HF)	0.88±0.41^b^

Wild-type and IVA-PLA_2_-knockout (KO) mice were fed normal (N) or high-fat (HF) diets for 16 weeks and fasted. The serum levels of PGE_2_ were measured (n = 3–5).

a *P*<0.05 versus wild-type mice fed normal diets.

b *P*<0.05 versus wild-type mice fed HF diets.

The elevation of serum aminotransferase level, as a marker of liver damage, is correlated with NAFLD [9]. In agreement with this, we show here that the serum level of alanine aminotransferase (ALT) was significantly higher in wild-type mice fed HF diets for 8 weeks than in wild-type mice fed normal diets, and the serum levels of both ALT and aspartate aminotransferase (AST) were markedly increased after 16 weeks of HF feeding (Table 1). However, no elevation of these aminotransferase levels was observed in IVA-PLA_2_-knockout mice even under HF dietary conditions. Considering the correlation of hepatic fat deposition with liver damage, it is likely that the protection against the HF diet-induced elevation of serum aminotransferase levels in IVA-PLA_2_-knockout mice is due to the reduced hepatic vacuolation and TG content.

**Figure 6:**
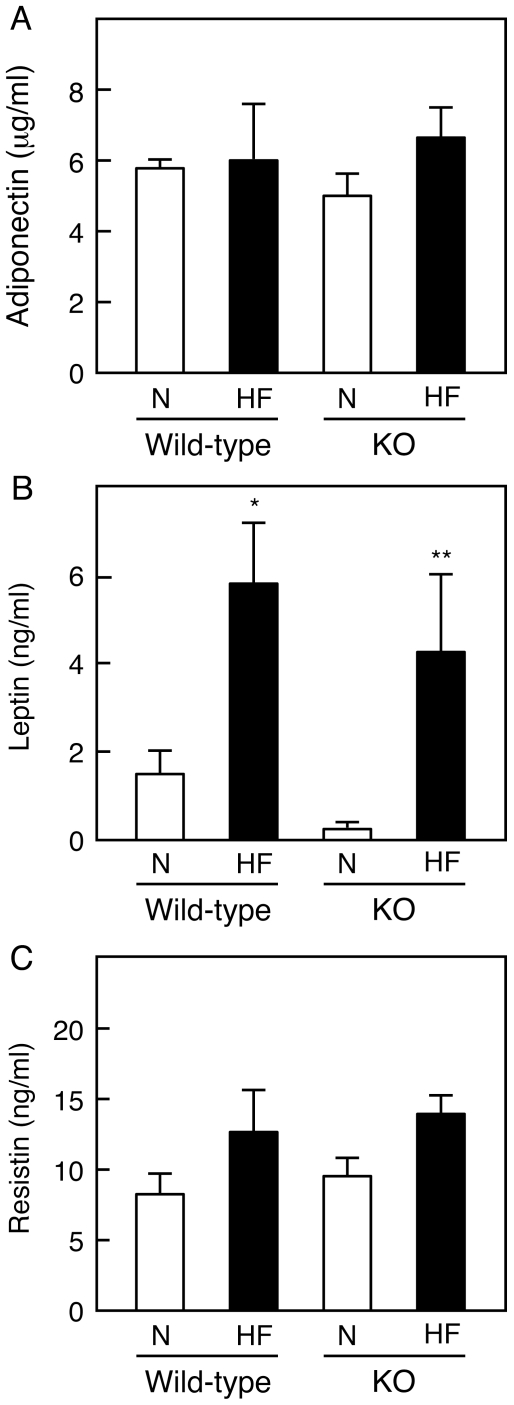
Serum levels of adipokines in wild-type and IVA-PLA_2_-knockout mice. Wild-type and IVA-PLA_2_-knockout (KO) mice were fed normal (N) or high-fat (HF) diets for 8 weeks and fasted. The serum levels of adiponectin (A), leptin (B), and resistin (C) were measured (n = 4–6). *P<0.05 versus wild-type mice fed normal diets, **P<0.05 versus IVA-PLA_2_-knockout mice fed normal diets. No significant differences in the serum levels of adipokines were detected between the two genotypes.

It is conceivable that changes in the levels of adipokines including adiponectin, leptin, and resistin influence the accumulation of intracellular lipids in the liver [10], [11]. Taking this into account, we determined the serum levels of adipokines in wild-type and IVA-PLA_2_-knockout mice fed normal or HF diets. As shown in Figure 6A, HF diets taken for 8 weeks did not affect the serum levels of adiponectin in wild-type and IVA-PLA_2_-knockout mice. In contrast, HF diets increased the serum levels of leptin in both genotypes, with no difference exhibited between the levels in the two groups (Figure 6B). Furthermore, no significant difference was observed in the serum levels of resistin among the four groups under our experimental conditions (Figure 6C).

## Discussion

Fatty liver with hepatocellular damage occurs in parallel with the accumulation of hepatic TG, which is associated with abdominal obesity. The present study showed that HF feeding of wild-type mice increased hepatocellular vacuolation, hepatic TG content, and serum aminotransferase levels with increases in adipose tissue mass and the number of hypertrophic adipocytes, indicating that HF diet-fed wild-type mice exhibited fatty liver damage with adipose fat deposition. The amounts of hepatic and adipose fats are regulated by several factors including FFA, insulin, adipokines, and PGs. Among these factors, PGs are generated by the cyclooxygenase pathway of the arachidonic acid cascade, the first step of which is mainly catalyzed by IVA-PLA_2_ [1]. Previous reports showed that PGE_2_ and 15-deoxy-PGJ_2_ accelerate the deposition of fats in hepatocytes and adipocytes [3], [5], [12]. Furthermore, the administration of PGE_2_ to rats increases hepatic TG content [4]. In addition to PGs, arachidonic acid metabolites of the 12-lipoxygenase pathway, such as 12-hydroxyeicosatetraenoic acid, are suggested to mediate inflammation occurring with fat deposition in adipose tissues [13]. Considering the role of IVA-PLA_2_ in the arachidonic acid cascade, it is possible that hepatic fat deposition could be ameliorated when the production of such arachidonic acid metabolites is suppressed by the inhibition of IVA-PLA_2_. Consistent with our expectations, the present study clearly showed that in IVA-PLA_2_-knockout mice with lower serum levels of PGE_2_, a lack of IVA-PLA_2_ alleviated the HF diet-induced hepatic fat deposition with hepatocellular damage, and reduced adipose accumulation. Our findings suggest that an inhibition of IVA-PLA_2_ prevents the HF diet-induced development of fatty liver damage. This notion is consistent with a recent observation that the administration of a cyclooxygenase-2 inhibitor to HF diet-fed rats prevents fat deposition in the liver and adipose tissues [14].

The present study further shows that HF diet-fed wild-type mice exhibited fatty liver damage with higher serum levels of phospholipids, lysophosphatidylcholine, and LDL. However, in IVA-PLA_2_-knockout mice, a deficiency of IVA-PLA_2_ reduced the HF diet-induced increase in these serum levels as well as fatty liver damage. These findings seem to indicate that the suppressed increase in LDL levels in IVA-PLA_2_-knockout mice fed HF diets probably results from suppressing the HF diet-induced development of fatty liver damage. In addition, the changes in the serum levels of phospholipids and lysophosphatidylcholine may be correlated with the serum levels of LDL.

Obesity with fat deposition in abdominal adipose tissues is implicated in the accumulation of hepatic TG through an increased influx of FFA into hepatocytes from hypertrophied adipocytes [7], [8], and/or through alterations of the secretion of adipokines including adiponectin and resistin that regulate, respectively, fat burning and fat storage in the liver [8], [10], [11], [15]. However, under our experimental conditions, wild-type mice on HF diets exhibited no change in the serum levels of not only FFA, adiponectin, and resistin, but also TG, glucose, or insulin, despite hepatic and adipose fat deposition. Thus, HF diet-fed wild-type mice developed fatty liver and adipose accumulation in the absence of significant features of metabolic disorders. This suggests that the HF diet-induced hepatic fat deposition is mediated in part by the direct deposition of dietary fats, as in the case of adipose tissues, in wild-type mice. Our recent [2] and the present studies showed that hepatic TG content was lower in IVA-PLA_2_-knockout mice than in wild-type mice under normal dietary conditions, suggesting a physiological role of IVA-PLA_2_ in the regulation of TG accumulation in the liver. Furthermore, HF diet-induced increase in hepatic TG content was partially suppressed in IVA-PLA_2_-knockout mice compared with wild-type mice, the reduced level being almost at the level observed in wild-type mice fed normal diets. It is, consequently, possible that since IVA-PLA_2_ is physiologically involved in the regulation of TG content in the liver, a lack of IVA-PLA_2_ somewhat prevents hepatic TG deposition even caused by excess dietary fat intake, thereby resulting in the alleviation of large hepatic vacuolation. It has been suggested that PGE_2_ plays a role in hepatocellular TG accumulation [3], [4]. In IVA-PLA_2_-knockout mice, serum PGE_2_ levels were indeed reduced. However, other IVA-PLA_2_ metabolites, such as other eicosanoids and lysophospholipids, are also probably reduced in IVA-PLA_2_-knockout mice. Therefore, IVA-PLA_2_ metabolites responsible for the accumulation of hepatic TG are unclear at present. Meanwhile, a recent study showed that an HF diet-induced increase in the mass of visceral adipose tissues precedes the development of fatty liver accompanied with no significant change in the serum levels of FFA, TG, or adiponectin in moderately obese mice [16]. Furthermore, fatty liver has been shown to occur through adipose accumulation with adipocyte hypertrophy and slightly decreased serum adiponectin levels, but without impaired glucose tolerance or any change in the serum levels of FFA, TG, glucose, insulin, or leptin in mice with an adipose-specific STAT3 (signal transducer and activator of transcription 3) disruption resulting in leptin resistance in adipocytes, even under normal dietary conditions [17]. This observation suggests that adipose accumulation and/or lower circulating adiponectin levels can cause the development of fatty liver in the absence of high dietary fats and metabolic disorders. Taking these findings and the notion mentioned above into account, the present results also suggest that the protection against the development of fatty liver damage in IVA-PLA_2_-knockout mice fed HF diets may be ascribable, in part, to the tendency towards a decreased adipose tissue mass with smaller adipocytes. However, to clarify the precise roles of IVA-PLA_2_ in the liver and adipose tissues, further studies with liver- or adipose-specific IVA-PLA_2_-knockout mice are needed.

Regarding the role of PGs in an increase in adipose tissue mass, PGE_2_ accelerates the deposition of intracellular TG by suppressing the production of cAMP that induces lipolysis through the activation of hormone-sensitive lipase in human and rat adipocytes [12]. Furthermore, 15-deoxy-PGJ_2_ endogenously generated in mature 3T3-L1 adipocytes participates in the accumulation of TG probably through the activation of peroxisome proliferator-activated receptor-γ [5]. These findings suggest roles for PGE_2_ and 15-deoxy-PGJ_2_ in the hypertrophy of mature adipocytes. In addition, PGI_2_ and 15-deoxy-PGJ_2_ play roles in the differentiation of preadipocytes into mature adipocytes resulting in adipocyte hyperplasia [12], [18]. In contrast, PGF_2α_ inhibits preadipocyte differentiation [19]. Thus, PGs exhibit various effects on adipogenesis *in vitro*. A previous study demonstrated that the differentiation of 3T3-L1 preadipocytes is inhibited by the knockdown of group VIA PLA_2_ or group VIB PLA_2_, other isozymes of intracellular PLA_2_ [20], suggesting the contribution of the isozymes to the generation of PGs responsible for preadipocyte differentiation. Recently, we reported that adipose tissue mass and adipocyte size are reduced in IVA-PLA_2_-knockout mice fed normal diets compared with wild-type mice [2], with similar results being observed in the present study. Furthermore, we show here that under the HF dietary conditions, IVA-PLA_2_-knockout mice with lower serum levels of PGE_2_ had lower adipose tissue mass and smaller adipocytes than wild-type mice, suggesting that the lower adipose tissue mass in IVA-PLA_2_-knockout mice fed HF diets probably results from the smaller adipocytes. Collectively, IVA-PLA_2_ may also be involved in adipose accumulation through the generation of PGs responsible for adipocyte hypertrophy.

The development of NAFLD is associated with the lower circulating levels of adiponectin [21], hepatic fat deposition being presumably due, in part, to the decreased fat burning effects of adiponectin in the liver. Consistent with this notion, a previous study showed that the administration of adiponectin to obese *ob/ob* mice, resulting in its higher concentration in serum, has beneficial effects on fatty liver [22]. This suggests that adiponectin itself or factors that regulate the production of adiponectin can be potent therapeutic molecular targets for the prevention of fatty liver. As inhibitory regulators of adiponectin production, PGD_2_, PGJ_2_, and 15-deoxy-PGJ_2_ are shown to suppress the expression of adiponectin in 3T3-L1 adipocytes [23]. In contrast, the serum levels of adiponectin decrease in lipocalin-type PGD synthase-knockout mice [24], suggesting a contribution of PGD_2_ to the production of adiponectin. Among the inhibitory and stimulatory effects of PGs, the former may be involved in the decrease in adiponectin level observed in obesity and NAFLD. In the present study, however, there was no difference between serum adiponectin levels in wild-type and IVA-PLA_2_-knockout mice, irrespective of the kind of diet, although the deficient mice were resistant to the HF diet-induced development of fatty liver damage. At present, the reasons for the lack of change in serum adiponectin levels in IVA-PLA_2_-knockout mice are unclear. Other PLA_2_ isozymes might be involved in the generation of PGs responsible for regulating the expression of adiponectin. Alternatively, a deficiency of IVA-PLA_2_ might result in decreases in the levels of both inhibitory and stimulatory PGs on the production of adiponectin. Meanwhile, the serum levels of leptin correlate positively with adipose accumulation [25]. Consistent with this, we show here that HF diets increased the serum levels of leptin with adipose accumulation in wild-type and IVA-PLA_2_-knockout mice. However, a deficiency of IVA-PLA_2_ did not affect the HF diet-induced increase in serum leptin levels. Considering the beneficial effects of leptin on fatty liver [26], [27], the changes in serum leptin levels under the HF dietary conditions probably do not explain the reduced fatty liver damage in IVA-PLA_2_-knockout mice. Previous studies showed that the expression of leptin is inhibited by PGD_2_, PGJ_2_, and 15-deoxy-PGJ_2_ in 3T3-L1 adipocytes [23], but stimulated by PGE_2_ in mouse adipose tissues in primary culture [28]. As in the case of adiponectin mentioned above, the possible involvement of IVA-PLA_2_ in the generation of PGs that regulate the production of leptin is unclear at present. To further clarify the role of IVA-PLA_2_ in the expression of adiponectin and leptin in adipocytes and colocalized inflammatory cells, experiments with preadipocytes and macrophages derived from IVA-PLA_2_-knockout mice are underway in our laboratory.

Resistin has been implicated in the pathogenesis of obesity-mediated insulin resistance [15]. Increased serum levels of resistin are related to the histological severity of NAFLD in human subjects [29]. A previous study demonstrated that hepatic TG content tended to be lower in resistin-deficient mice [30]. These findings suggest that higher serum levels of resistin are a cause of the development of fatty liver. In the present study, no significant difference was observed in the serum levels of resistin in wild-type and IVA-PLA_2_-knockout mice. This observation is consistent with the lack of changes in the serum levels of insulin and glucose in both genotypes under the HF dietary conditions. Under our experimental conditions, fatty liver damage in wild-type mice probably develops in the absence of insulin resistance.

In summary, the present study demonstrates that a deficiency of IVA-PLA_2_ protected mice against the HF diet-induced development of fatty liver damage with adipose accumulation. The alleviation of fatty liver damage is probably associated with the decreased generation of IVA-PLA_2_ metabolites including PGs. To our knowledge, this is the first report to suggest the possible involvement of IVA-PLA_2_ in hepatic fat deposition progressing to NAFLD under HF dietary conditions.

## Materials and Methods

### Mice

Homozygous IVA-PLA_2_-knockout mice with a C57BL/6 background (more than N19 generations) were generated by the cross breeding of heterozygous IVA-PLA_2_-knockout mice, which were a generous gift from Dr. T. Shimizu and Dr. N. Uozumi (Department of Biochemistry and Molecular Biology, the University of Tokyo) [31]. Male IVA-PLA_2_-knockout and wild-type mice (6 weeks of age) were kept under a 12 h light/dark cycle, and given normal (5.3% fat) or HF (D12108: 20% fat and 1.25% cholesterol, Research Diets Inc.) diets for 8 or 16 weeks. For measurement of food consumption, mice were housed individually in cages with wire-screen floors and no bedding. Preweighed food was placed in food cups attached to the cage floor with spring clips. Food intake was then measured daily for 3 days before the end of HF feeding period by weighting the food with careful monitoring of any spillage. This study was approved by the Experimental Animal Research Committee at Kyoto Pharmaceutical University.

### Histological Analysis

The animals were fasted for 20 h, and body weights were measured before the collection of the blood and tissues. Mice were deeply anesthetized with pentobarbital (0.3 mg/g body weight, intraperitoneal administration). After the blood was collected from the inferior vena cava, mice were perfused transcardially with saline using a peristaltic pump. The liver and epidermal fat pads were isolated and weighed. The isolated tissues were fixed in 10% buffered formaldehyde and embedded in a paraffin block for microtome slicing into 4-µm thick sections. The tissue sections were deparaffinized and stained with hematoxylin-eosin or periodic acid-Schiff. The stained sections were photographed using a microscope (model IX71, Olympus, Tokyo, Japan) with a digital camera.

### Measurement of Biochemical Parameters and Analysis of Lipoproteins in Serum

Collected blood samples from the inferior vena cava were placed for 30 min at room temperature, and the supernatants were obtained as serum after centrifugation. The serum levels of TG, glucose, ALT, and AST were measured with an automatic analyzer (DRI-CHEM 7000, Fujifilm Medical, Tokyo, Japan). The serum levels of FFA, phospholipids, lysophosphatidylcholine, insulin, adiponectin, leptin, and resistin were quantified with commercial assay kits (Wako Pure Chemical Industries, Osaka, Japan; Sceti Medical Labo K.K., Tokyo, Japan; R&D Systems, Inc., Minneapolis, MN). For the determination of the serum levels of PGE_2_, the serum from mice were collected and treated with indomethacin (10 µg/ml). PGE_2_ was extracted from the samples by solid phase extraction on a C18 cartridge. The concentrations of PGE_2_ were analyzed with a commercial ELISA kit (R&D Systems, Inc.) according to the manufacturer's instructions. Lipoproteins in the serum were analyzed with a liquid chromatography system equipped with a reactor for a postcolumn reaction to automatically measure the total amounts of cholesterol of separated lipoproteins.

### Measurement of Hepatic TG

The liver (100 mg wet tissue) was homogenized in an ice-cold 0.05% butylhydroxytoluene solution. After lipids were extracted from the liver according to the method of Folch *et al*. [32] with a slight modification, TG content in each sample was measured with a commercial assay kit (Wako Pure Chemical Industries, Osaka, Japan).

### Statistical Analysis

Values are expressed as the mean ± SEM. Data were analyzed with Student's *t*-test. *P*<0.05 was considered statistically significant.
